# Restless Legs Syndrome in Adult Primary Care

**DOI:** 10.7759/cureus.90090

**Published:** 2025-08-14

**Authors:** Ankit Mathur, Abid Bhat, Ashraf Gohar

**Affiliations:** 1 Sleep Medicine, University of Missouri-Kansas City School of Medicine, Kansas City, USA

**Keywords:** alpha-2-delta ligands, augmentation, dopamine agonists, iron deficiency, primary health care, restless legs syndrome, willis-ekbom disease

## Abstract

Restless Legs Syndrome (RLS) or Willis‑Ekbom Disease is a sensorimotor condition marked by an irresistible need to move the legs, typically accompanied by uncomfortable sensations that peak during periods of rest and disrupt nightly sleep. Early identification in primary care is essential, as timely intervention can dramatically improve the patient's quality of life. Diagnosis relies on a focused clinical history, guided by targeted questions that explore symptom timing, triggers, and relief measures. Management begins with non‑pharmacological strategies, such as optimizing sleep hygiene and correcting iron deficiency, before progressing to pharmacologic options like gabapentinoids or dopamine agonists when needed. By combining lifestyle modifications with tailored medication plans, clinicians can effectively reduce symptoms and improve sleep quality.

## Introduction and background

Restless Legs Syndrome (RLS), also known as Willis-Ekbom Disease, is a sensorimotor disorder characterized by an irresistible urge to move the legs. This is often accompanied by uncomfortable sensations felt in the limbs such as tingling, crawling, or itching [1]. RLS has a circadian pattern, and symptoms usually occur or worsen in the evening or at bedtime [2,3]. The symptoms occur and typically worsen during periods of rest and are alleviated by movement [4]. RLS affects approximately five to 10% of adults in the United States, with a higher prevalence observed in females and older adults [5,6]. A systematic review of RLS frequency in children found that 1.9% of those from eight to 11 years of age and 2% of those from 12 to 17 years of age reported symptoms of RLS [7]. The condition tends to become apparent earlier in individuals with a family history of RLS, but its severity increases with age. About one-third of individuals with RLS experience symptoms severe enough to require medical intervention [8,9]. About 21-57% of individuals with RLS report some arm sensation; however, isolated arm involvement is extremely rare [1,2]. Given the high prevalence of RLS, primary care physicians should familiarize themselves with the condition and the importance of its management [4].

## Review

Pathophysiology and etiology 

The pathophysiology of RLS remains incompletely understood. It likely involves multifaceted interactions between genetic factors, iron deficiency in the brain, and disturbances in dopaminergic neurotransmission. RLS is a genetically complex disorder, with 50-60% of individuals with RLS having a family history [3,10]. Patients with hereditary RLS may experience an earlier onset of disease [10]. Several genetic loci, including MEIS1 and BTBD, have been identified as risk loci, but MEIS1 is believed to be the strongest risk factor for RLS. MEIS1 appears to play an important role in brain iron metabolism [11].

Central iron deficiency appears to be a key factor in RLS pathophysiology. Imaging and autopsy studies reveal decreased iron concentrations specifically within the CNS regions involved in motor and sensory processing, mainly the substantia nigra and basal ganglia, even in patients without systemic iron deficiency [12,13]. Reduced CNS iron is believed to disrupt dopamine synthesis and metabolism. Additionally, decreased iron availability in the brain may lead to altered dopamine receptor density and sensitivity [14,15]. It has been suggested that the A11 dopamine system, which descends to the spinal cord, could be involved in RLS, acting through the D3 dopamine receptors [16]. ​​​​​​Disturbances in CNS dopamine signaling further support the theory on why dopaminergic agents frequently alleviate symptoms, and dopamine-blocking agents exacerbate or induce symptoms [4,17]. Iron deficiency in the brain of rodents is linked with changes in adenosinergic transmission, and downregulation of the adenosine A1 receptor (A1R) is the main finding. This downward regulation of A1R leads to hypersensitive striatal glutamatergic terminals and facilitation of striatal dopamine release [18]. 

Clinically, RLS is classified as either primary (idiopathic) or secondary. Primary RLS typically has a strong genetic component, about 50%, and tends to present earlier in life. These symptoms tend to worsen with age [3]. Secondary RLS is associated with various underlying medical conditions, including systemic iron deficiency, chronic kidney disease (CKD), diabetes mellitus, peripheral neuropathies, pregnancy, and use of specific medications such as antihistamines, antidepressants, and antipsychotics [16]. In pregnancy, up to 19% of women experience RLS symptoms, most commonly in the third trimester, although symptoms usually resolve postpartum [19,20]. In CKD, predominantly end-stage renal disease requiring dialysis, RLS prevalence reaches up to 50%, significantly impacting quality of life, sleep quality, and overall health [21].

Understanding these complex etiological and pathophysiological factors provides a comprehensive foundation for clinicians to recognize, diagnose, and effectively manage RLS.

Iron deficiency and RLS

Iron deficiency, has been closely associated with RLS, as serum ferritin levels (<75 ng per ml) in adults have been shown to correlate with increased severity of symptoms [1]. Additionally, ferritin levels in the cerebrospinal fluid are often lower in patients with RLS, suggesting iron deficiency in the brain, even in the absence of systemic iron deficiency [12]. It is also important to assess transferrin saturation. A transferrin saturation below 20% may further support the diagnosis of iron-deficient RLS, even when ferritin levels are borderline [19]. Primary care providers should routinely screen patients presenting with RLS symptoms for iron deficiency by obtaining serum ferritin and transferrin saturation levels, particularly given that early identification and correction of iron deficiency can help alleviate symptoms and reduce the need for long-term medication use [22]. Intervention in patients with iron deficiency may also prevent symptom progression and improve the overall quality of life [22].

Clinical presentation

Patients with RLS characteristically describe a compelling urge to move their legs, commonly accompanied by uncomfortable sensations such as crawling, tingling, burning, or itching [1,2]. These sensations are typically challenging for patients to describe and are often seen to be coming from deep within the legs rather than superficially [2]. Leg pain is typically not a symptom of RLS. In case it is present, conditions including neuropathy or radiculopathy must be considered. Symptoms typically arise or worsen during periods of rest, particularly during prolonged sitting or lying down, making activities of prolonged rest particularly challenging [1,3]. Examples include lying down in bed to sleep, driving a car, and flying in an airplane.

Movement usually provides immediate, although temporary, relief from symptoms. Common ways patients relieve their symptoms include pacing, stretching, massaging, or shaking their legs [2,4]. Dopamine has a clear circadian activity pattern, decreasing in the evening and night and increasing in the morning. This explains the circadian pattern of RLS, characterized by intensified symptoms during evening and nighttime hours, that often leads to sleep disturbances such as delayed sleep onset and frequent nocturnal awakenings [1,3,4]. 

As a result of poor sleep quality, patients frequently experience daytime symptoms, including chronic fatigue, tiredness, impaired concentration, irritability, and diminished cognitive function [4,5]. Such symptoms can have a worsening impact on quality of life, reducing overall productivity, negatively affecting mood, and impairing social and occupational functioning [4,5]. 

RLS presentations vary widely, ranging from mild intermittent symptoms, less than twice a week, to severe chronic symptoms that occur daily or near-daily and often require ongoing pharmacological intervention [5,6]. Recognizing the variability of signs and symptoms is crucial for providers to better manage patients and enhance outcomes [1,4]. 

Diagnostic criteria and evaluation

Patients must meet the diagnostic criteria under the International Classification of Sleep Disorders, Third Edition, Text Revision (ICSD-3-TR) for RLS to establish an accurate diagnosis. The four criteria are urge to move the legs, symptoms start or worsen when the person is at rest or inactive, getting up and moving helps relieve symptoms temporarily, and lastly, symptoms worsen in the evening or night [1]. Moreover, the above features cannot be caused by conditions that mimic RLS symptoms. It is important to ask questions regarding the sleep/wake cycle and signs of sleepiness in patients with symptoms of disturbed sleep and daytime fatigue, as this is another criterion for RLS diagnosis [2,23]. Polysomnography (PSG) is not normally indicated for the diagnosis of RLS but can be helpful in demonstrating significant objective sleep anomalies. Consistent findings in PSG include increased sleep onset latency and a higher arousal index. Periodic limb movements (PLMs)>five/hour occur in 70-80% of adults with RLS on a single-night testing [13]. Helpful lab tests include ferritin levels (<75 ng/ml) and serum chemistries to rule out uremia and diabetes mellitus [14]. The physical exam is usually normal in patients with RLS.

Differential diagnosis

Several conditions, including akathisia, nocturnal leg cramps, vascular conditions, arthritis, and attention-deficit/hyperactivity disorder can mimic the symptoms of RLS, requiring careful clinical evaluation and diagnostic testing to eliminate alternative diagnoses. Akathisia, often resulting from medications such as antipsychotics or other dopamine-blocking agents, is characterized by an irresistible need to move, but typically lacks the sensory discomfort characteristic of RLS [24]. Unlike RLS, symptoms of akathisia do not display a consistent circadian pattern or consistent worsening during periods of rest [3,24].

Nocturnal leg cramps, another condition in the differential diagnosis, present with sudden, involuntary, and painful muscle contractions. Unlike RLS, these muscle cramps often have a sudden onset, typically affect certain muscle groups such as the calf muscles, and may not be relieved by movement [3,17].

Peripheral neuropathy, which has multiple etiologies including diabetes mellitus, alcohol abuse, nutritional deficiencies, and autoimmune conditions, can also mimic RLS by producing sensory disturbances such as burning, tingling, numbness, and sharp pain [4,17]. However, these neuropathic symptoms may be persistent, occurring at any time, and are often less responsive to voluntary movement than RLS [17].

Vascular conditions, including peripheral arterial disease (PAD) and deep vein thrombosis (DVT), can present similarly with limb discomfort, particularly at rest. PAD often causes claudication symptoms, portrayed by exercise-induced muscle pain that resolves with rest, a pattern opposite to that of RLS. DVT typically presents acutely, with unilateral limb swelling, redness, warmth, and pain, and symptoms do not improve with movement or pacing [4,24]. Detailed clinical history, physical examination, and appropriate diagnostic investigations, including laboratory tests (e.g., serum ferritin, glucose levels) and imaging, when necessary, help differentiate these conditions from RLS.

Management and treatment approaches

The severity of RLS varies from patient to patient. There are both non-pharmacologic and pharmacologic treatments to manage RLS.

Lifestyle Modification

The first steps would be to limit alcohol, caffeine, and tobacco products, which can help improve symptoms. It may be helpful to avoid medications that can worsen symptoms, such as antihistamines, lithium, tricyclic antidepressants (TCAs), selective serotonin reuptake inhibitors (SSRIs), and dopamine antagonists [25]. Bupropion is one of the few antidepressants that does not exacerbate RLS, making it a preferred option for patients requiring antidepressant therapy. Increasing aerobic activity has also been shown to improve symptoms compared to sedentary patients [26,27]. 

Iron Supplementation

Patients with RLS may need to optimize their iron stores, either by increasing iron-rich foods or starting oral or intravenous (IV) iron supplementation if ferritin levels are below 75 ng/ml or transferrin is <20% [1]. For most patients, because oral iron is easier to administer, initial therapy with an oral regimen such as ferrous sulfate taken with vitamin C to increase absorption is suggested. Oral iron should not be taken at the same time as calcium supplements. IV iron therapy is generally reserved for patients with intolerance to oral iron preparations, malabsorption state, persistence of symptoms despite a trial of oral iron, and those with a need for a more rapid response due to severity of symptoms.​​ All IV iron formulations must be administered in a setting where treatment for anaphylaxis is available.

Pharmacologic Medications

Traditionally, the primary pharmacologic treatment for RLS relied on dopamine agonists, including pramipexole, ropinirole, and rotigotine [25]. These medications were effective in decreasing RLS symptoms and improving sleep quality. However long-term use frequently leads to augmentation and a worsening of symptoms characterized by earlier onset and increased severity, which spread to other body parts [26]. Augmentation is particularly concerning because it often results in a cycle of rising medication doses, ultimately worsening symptom control and complicating long-term management [28]. Carbidopa/levodopa, once used for intermittent symptoms, carried an even higher risk of augmentation and is now generally avoided [25]. In addition, dopamine agonists have been associated with worsening symptoms of obsessive-compulsive disorder and the emergence of impulse control disorders. As a result, their use should be avoided or approached with caution in patients with RLS and coexisting obsessive-compulsive disorder [29]. Recent updates in RLS management reflect a shift away from dopamine agonists in favor of gabapentinoids, which have a lower risk of augmentation [25]. The recent American Academy of Sleep Medicine (AASM) guidelines suggest against the use of clonazepam. 

The AASM now strongly recommends gabapentin enacarbil, gabapentin, and pregabalin as first-line options for patients with moderate-to-severe symptoms [22]. Table 3 summarizes the major pharmacologic treatment options for RLS, including their indications, common side effects, and guideline-based recommendation to aid clinical decision-making. 

**Table 1:** Pharmacologic treatments for Restless Legs Syndrome (RLS)

Treatment Class (Examples)	Indications	Common side effects	Highlights of the American Academy of Sleep Medicine (AASM) guidelines
Iron therapy (oral or IV iron)	Ferritin <75 ng/mL or transferrin saturation <20%; evidence of iron deficiency [25]	Oral: gastrointestinal upset, constipation IV: infusion reactions, rarely anaphylaxis [26]	Initiate when iron deficiency confirmed; reassess iron status after 3 months [25]
Gabapentinoids (e.g., gabapentin enacarbil, pregabalin)	Moderate-to-severe RLS; especially useful with insomnia, anxiety, neuropathic pain, or concerns regarding augmentation [25]	Dizziness, somnolence, peripheral edema, weight gain [28]	First-line therapy; preferred due to lower risk of augmentation compared to dopamine agonists [25]
Dopamine agonists (e.g., pramipexole, ropinirole, rotigotine patch)	Moderate-to-severe RLS without prominent insomnia, anxiety, or neuropathic pain; caution due to augmentation risk [26]	Nausea, dizziness, hypotension, impulse-control disorders, augmentation [26]	Second-line therapy; use lowest effective dose, and closely monitor for augmentation. Transdermal patches (rotigotine) available [25,28]
Refractory therapies (opioids or benzodiazepines)	Severe refractory RLS unresponsive to gabapentinoids or dopamine agonists; benzodiazepines primarily for insomnia [28]	Opioids: sedation, dependency, constipation, respiratory depression (rare) Benzodiazepines: sedation, cognitive impairment, dependency risk [26,28]	Third-line/adjunctive; opioids (e.g., oxycodone) reserved for severe, refractory symptoms; benzodiazepines (e.g., clonazepam) for associated insomnia or anxiety. Use cautiously [25,28]

These medications have demonstrated significant improvements in disease severity and sleep quality while maintaining a good safety profile. However, both dopamine agonists and gabapentinoids are generally not considered safe during pregnancy, and non-pharmacologic strategies such as iron supplementation (if ferritin is <75 ng/mL), massage, exercise, and good sleep hygiene are recommended as first-line management for pregnant patients with RLS [28].

For patients with refractory RLS, treatment strategies have expanded to include alternative pharmacologic and neuromodulatory options. While opioids remain an option in refractory cases, they should be prescribed carefully due to potential dependency risks [28]. Initial use of lower potency opioid agents and escalation towards higher potency opioids is the preferred approach. Additionally, high-frequency peroneal nerve stimulation has shown promise as a non-pharmacologic intervention for patients with persistent symptoms [30]. These updates highlight a more individualized treatment approach, minimizing long-term risks associated with dopamine agonists while increasing therapeutic options for primary care providers managing RLS [28]. If patients continue to have refractory cases for RLS, further testing may be required and appropriate referral to Sleep Medicine or Neurology may be considered. 

## Conclusions

Primary care physicians play a crucial role in recognizing, diagnosing, and managing RLS. Combining focused sleep-related questions and symptom screening into routine patient evaluations is essential due to the high prevalence and significant quality of life impacts related to this disorder. Successful treatment relies on careful listening to patient experiences, customized treatment plans, and ongoing patient education. Given the chronic nature of RLS, physicians should begin with non-pharmacologic interventions and lifestyle adjustments whenever possible, including attention to sleep hygiene and careful review of medications that might worsen symptoms. In cases requiring medication, clinicians must thoughtfully weigh therapeutic benefits against potential side effects, regularly monitoring patients to adjust treatment accordingly. Encouraging patient self-awareness and symptom tracking can empower individuals, promoting better adherence to treatment and improved long-term outcomes. Overall, effective management requires a patient-centered approach, thorough assessment, and cooperative follow-up to ensure lasting symptom relief and enhanced quality of life.
